# Study protocol of the CAREST-trial: a randomised controlled trial on the (cost-) effectiveness of a CBT-based online self-help training for fear of cancer recurrence in women with curatively treated breast cancer

**DOI:** 10.1186/s12885-016-2562-0

**Published:** 2016-07-25

**Authors:** Sanne Jasperine van Helmondt, Marije Liesbeth van der Lee, Jolanda de Vries

**Affiliations:** 1 Scientific Research Department, Helen Dowling Instituut, Bilthoven, The Netherlands; 2 Center of Research on Psychology in Somatic diseases (CoRPS), Department of Medical and Clinical Psychology, Tilburg University, Tilburg, The Netherlands; 3 Department of Medical Psychology, St Elisabeth Hospital, Tilburg, The Netherlands

**Keywords:** Fear of cancer recurrence, Breast cancer, RCT, Self-help, Online, CBT, Cost-effectiveness

## Abstract

**Background:**

One of the most prevalent long-term consequences of surviving breast cancer is fear of cancer recurrence (FCR), which is associated with higher (mental) healthcare costs and lower surveillance rates. The majority of breast cancer survivors report a need for professional help in dealing with FCR. An easy-accessible and cost-effective evidence‐based psychological intervention for reducing FCR is lacking. In the current study an online self-help training to reduce FCR will be evaluated. In addition, the secondary aim of this study is to identify factors that predict whether women can benefit from the online self-help training or not.

**Methods/Design:**

A multi-centre, parallel-groups, randomised controlled trial will be conducted to evaluate the (cost-) effectiveness of the CAREST-trial. A sample of 454 women with curatively treated breast cancer will be recruited from 8 hospitals in the Netherlands. Participants will be randomised to the intervention or usual care group (1:1). Self-report measures will be completed at baseline, 3 (post-intervention), 9, and 24 months. Primary outcome is FCR severity; secondary outcomes are healthcare costs, health status, and psychological distress. The online tailored self-help training “Less fear after cancer” is based on cognitive behavioural therapy and consists of 2 basic modules (psycho-education; basic principles of cognitive behavioural therapy) and 4 optional modules (rumination; action; relaxation; reassurance) to choose from. Each module consists of an informative part (texts, videos, audio files) and a practical part (exercises). For every patient, the intervention will be available for three months. Personal online support by an e-mail coach is available.

**Discussion:**

Online self-help training may be an easy-accessible and cost-effective treatment to reduce the impact of FCR at an early stage in a large group of breast cancer survivors. A strength is the 24 months follow-up period in the health economic evaluation. The results of the study will provide information on the possible strengths and benefits of online self-help training for FCR in breast cancer survivors.

**Trial registration:**

This study is registered at the Netherlands Trial Register (NTR4119, date registered: August 15, 2013).

## Background

Due to earlier diagnosis and improved medical treatments, the number of women living with breast cancer is rising. In the Netherlands, the prevalence of breast cancer has been estimated to rise from 100.000 in 2000 to 140.000 in 2020 [1]. Similarly, in the USA, the 5-year relative survival rate of women with breast cancer improved from 75 % in 1975 to 90 % in 2009 [2]. One of the most prevalent long-term consequences of surviving cancer is fear of cancer recurrence (FCR), which can be defined as the fear or worry of the possibility that the cancer will return or progress in the same organ or in another part of the body [3]. Although the majority (82 %) of long-term breast cancer survivors reported low levels of FCR, a considerable amount of women (17 %) reported moderate to high FCR [4]. In younger (age 18–45) early-stage breast cancer survivors, 70 % of participants report moderate to high levels of FCR [5]. Younger age was also found to be associated with a higher intensity of FCR [5, 6]. Moderate to high levels of FCR have also been found in 29 % of women with ductal carcinoma in situ or early invasive breast cancer two years after diagnosis [7].

Findings on the long-term course of FCR are still unclear [8]. Thewes and colleagues [9] found longer time associated with reduced FCR in younger cancer patients. Several other studies found no significant relationship between time since diagnosis and FCR [3, 10–15]. Elevated levels of FCR represent a continuing problem in cancer patients, up to sixteen years after diagnosis [3, 10–15]. For example, long-term breast cancer survivors (5–12 years after diagnosis) report many factors that can trigger FCR, such as hearing about cancer, unclear bodily complaints, environmental triggers (e.g., on television, the internet, newspapers, and magazines; and visiting a doctor [16]. Women encounter these triggers about twice a month [16].

Thus, FCR is a common and continuing problem in breast cancer survivors. Women cope with FCR in different ways. Elevated levels of FCR has been found to be associated with higher frequency of unscheduled visits to the general practitioner, larger number of outpatient and emergency room visits, and more use of health care services [5, 16, 17]. Higher levels of FCR may also lead to avoiding forms of cancer screening and medical control visits [5, 16, 17]. Moreover, FCR may lead to various types of (compulsive) self-examining [5, 16, 17]. Therefore, it is not surprising that preliminary evidence shows that FCR is associated with higher healthcare costs and lower surveillance rates, which may compromise health outcomes [5].

Depending on the coping mechanisms used by breast cancer survivors to deal with their FCR, FCR also has a considerable impact on their lives. One of the most significant effects of FCR is the negative impact on quality of life [4, 18, 19]. Furthermore, FCR may have a correlation with distress, intrusive thoughts, avoidance, hyperarousal, psychological disorders (e.g., depression, anxiety symptoms, posttraumatic stress disorder), and fatigue [3, 4, 13, 14, 20]. Subsequently, FCR is associated with higher mental health costs [5, 19].

Cancer survivors frequently identify FCR as a major concern and 20 to 79 % of them report to have a need for professional help coping with FCR [19, 21]. About 30 % of cancer survivors have indicated that there is no support for them in dealing with FCR [22]. Reasons for this include lack of recognition, lack of trained mental health professionals, insurance coverage and cost issues, and geographical distance from providers [22, 23]. Considering the increasing prevalence of breast cancer, increasing healthcare costs, and the lack of professional help for FCR in a large group of cancer survivors [1, 2, 5], there is an urgent need for easy-accessible and cost-effective evidence‐based psychological interventions for reducing FCR.

Knowledge on treatment of FCR is limited. Simard and colleagues [19] found only five face-to-face group interventions (cognitive behaviour therapy, supportive-experiential therapy, mindfulness-based stress reduction, and emotion regulation) to reduce FCR. Furthermore, Völker and colleagues [24] suggest a stepped-care model for treating FCR. Normalization, psycho-education and self-management are the first steps in this model [24]. Normalization helps patients to understand that fear is a normal reaction which can be helpful in some situations. Psycho-education is necessary, because cancer survivors experience many bodily symptoms (such as fatigue, new aches and pains, muscle tension, joint stiffness, feeling of weakness, indigestion, and other physical symptoms) that can easily be misinterpreted as symptoms of recurrence [16]. Anxiety itself also can cause several bodily symptoms (such as increased heart rate, shortness of breath, chest pressure, sweating, dry mouth, dizziness, feeling of weakness, muscle tension, and indigestion), which may increase other bodily symptoms and therefore reinforce FCR. In extreme cases, FCR has been associated with the development of anxiety disorders [8]. Misinterpretation of bodily symptoms can lead to negative thinking, which causes somatic amplification. Because of this somatic amplification, patients focus even more on their bodily symptoms, leading to a negative emotional spiral. With psycho-education, patients gain more knowledge about the bodily mechanisms of fear. This knowledge can help them to break the negative spiral. Ziner and colleagues [25] found that breast cancer survivors with high self-efficacy in dealing with concerns related to breast cancer after treatment, had lower FCR and that self-efficacy may have a protective effect in these women. Therefore, training in self-efficacy may reduce FCR [24–26]. If these first steps of treatment for FCR turn out to be insufficient, cognitive behavioural therapy (CBT) should be the next step in the treatment of FCR [24, 27, 28]. Furthermore, acceptance focused therapies, such as Mindfulness Based Stress Reduction (MBSR), Mindfulness Based Cognitive Therapy (MBCT) and Acceptance and Commitment Therapy (ACT) may be included in treatment for FCR [24].

In order to reach the growing group of breast cancer survivors experiencing FCR, a CBT-based online self-help intervention (including normalization, psycho-education and self-management) may be an appropriate and accessible way to offer psychological treatment to patients for several reasons. First, self-help without support was the second most preferred type of supportive care by cancer patients (14–28 %), after individual professional counseling [21]. Furthermore, there is a growing body of evidence that internet interventions can improve psychological well-being in cancer patients [23]. Online self-help interventions have several advantages, including convenience (such as working on the self-help training in the evening when there are no competing demands), ability to proceed at one’s own pace to master the material, low cost, greater privacy and confidentiality, more comfort, the intervention content can be updated quickly, greater accessibility (for example for those living in rural areas), time- and cost-effective, and where waiting lists are long [23, 29–31]. Convenience and working in their own pace may be particularly attractive for young women, who often are still working and have young children [5]. Given the high prevalence of FCR in young breast cancer survivors, online self-help training may be an effective and attractive way to reach these young women [5]. Moreover, an informational self-management intervention may reduce psychological distress (feelings of tension, anger and depression) in high risk patients who perceive little control and much illness uncertainty [32]. Thus, an online self-help intervention with psycho-education may empower patients. Disadvantages of online self-help interventions are internet access constraints, technical difficulties, high drop-out rates, poor adherence, safety issues, limited personal interaction, and not being able to detect more complicated issues and non-verbal or verbal clues in patients [23, 31, 33, 34]. Furthermore, online cognitive behavioural interventions for anxiety have been found to be effective for anxiety disorders and anxiety symptoms [30, 35]. Online cognitive behavioural self-help interventions for anxiety have been found to be at least as good as face-to-face treatment in several studies, but other studies found small effects favoring face-to-face treatment [29, 34–37]. Self-help interventions may be specifically effective, if based on a theoretical model such as CBT [38]. In conclusion, CBT-based online self-help interventions are promising and may provide effective, acceptable, and practical health care for those who may otherwise remain untreated. Because online self-help training may be both easily accessible and cost-effective, in the current study an online CBT-based self-help training to reduce FCR after breast cancer treatment will be evaluated.

### Aims

The primary aim of the CAncer REcurrence Self-help Training [CAREST] randomised controlled trial is to evaluate the effectiveness and cost-effectiveness of the online CBT-based self-help training “Less fear after cancer” [in Dutch: “Minder angst bij kanker”] in reducing FCR in women with curatively treated breast cancer (breast cancer survivors), compared to usual care. Specific hypotheses are that:

1. FCR severity reduces more in the online self-help condition compared to the usual care condition between baseline and follow-up (3 months). This effect will sustain 9 and 24 months after baseline. Small effect sizes are expected.
2. Health care costs reduce more in the online self-help condition compared to the usual care condition between baseline and 24 months after baseline.
3. Psychological distress reduces more in the online self-help condition compared to the usual care condition between baseline and 3 months after baseline. These effects will sustain 9 and 24 months after baseline.

Online self-help for FCR is not expected to be effective for all participants. Several factors, such as level of FCR, psychological distress, coping strategies, and perceived self-efficacy may predict if women can benefit from the online self-help training. Therefore, the secondary aim is to identify factors that predict whether women can benefit from the online self-help training or not.

4. With regard to prediction of treatment effect we will explore the following hypotheses:
  - *Baseline fear of recurrence severity*. In participants with low scores on FCR severity there is no need for psychological help, these participants are expected not to use the online self-help training or to drop-out. In participants with moderate scores on FCR severity a modest effect of the online self-help training is expected. In participants with high scores on FCR severity there will be a modest effect of the self-help training and additional need for psychological help or guidance by a therapist.
  - *Psychological distress*. In participants with high scores on psychological distress extra psychological help will be needed, because psychological distress may interfere with self-management capacity. Participants with moderate scores on psychological distress will have enough motivation for self-help training. Participants with low scores on psychological distress are expected to have minimal motivation for self-help training and thus a low treatment effect.
  - *Level of functioning impairment*. In participants with high scores on level of functioning impairment extra psychological help will be needed. Participants with moderate scores on level of functioning impairment will have motivation for self-help training. Participants with low scores on level of functioning impairment will have minimal motivation for self-help training.
  - *Coping strategies*. The relation between coping and treatment effect will be explored.
  - *Psychosocial problems and risk factors*. If participants score high in many problem domains extra help may be needed. Moreover, high scores on trait anxiety and low scores on social support indicate that extra psychosocial help may be needed.
  - *Perceived self-efficacy for online self-help.* Participants with low scores on perceived self-efficacy for online self-help may have poor self-management skills. Treatment effect for these participants will be minimal and extra support may be needed. For participants with moderate and high scores on perceived self-efficacy for online self-help, the training which is being investigated will be appropriate and will lead to moderate treatment effect.
  - *Socio-demographic variables* with respect to treatment effect will be explored. For example, for young women and women with a partner, online self-help training may be more effective than older participants or those without a partner. Also, for medium to highly educated women the self-help training may be more effective.
  - *Medical variables* with respect to treatment effect will be explored.

## Methods/Design

The CAREST-study design and intervention will be reported in accordance with the CONSORT statements for eHealth interventions [39] and parallel group randomised trials [40], the SPIRIT 2013 statement [41, 42], and in accordance with the recommendations and guidelines for internet intervention research in psycho-oncology [23].

### Study design

The CAREST-study is a multi-centre, randomised controlled trial, comparing online self-help training with care as usual in breast cancer survivors. A sample of 454 women with curatively treated breast cancer will be recruited from 8 hospitals scattered over the Netherlands. The participating hospitals are the Maasstad hospital in Rotterdam, St Antonius hospital in Utrecht, Admiraal de Ruyter hospital in Vlissingen, Reinier de Graaf hospital in Delft, Antonius hospital in Sneek, St. Elisabeth hospital in Tilburg, Catharina hospital in Eindhoven, and UMCG in Groningen (all situated in the Netherlands). After completion of the baseline measure, women will be randomised to either the online self-help or control group. Follow-up assessments are at 3 months (post-intervention), 9 months, and 24 months after baseline. Additionally, every 3 months participants will be asked to fill out a short measure about their healthcare use. Two reminders will be sent by e-mail one and two weeks after the first invitational e-mails. Eventually, participants who do not complete the questionnaires will receive a phone call from the researcher to remind them.

### Participant eligibility

Women are eligible to participate if they had a diagnosis of breast cancer 1–5 years ago; have no signs of local or regional recurrence or metastatic disease; are capable of filling out questionnaires in Dutch; if their age at disease onset was 18 years or older; and if they have access to a computer with an internet connection. There are no exclusion criteria.

### Recruitment settings and procedure

Patients will be recruited in two ways. First, four hospital sites are expected to recruit participants through oncology nurses, nurse practitioners, or oncologists, who will ask eligible patients to participate in the study. These patients will be informed about the study during their regular check-up at the outpatient clinic. When women show interest in participating in the study, they receive a comprehensive information letter. In three hospitals patients will be phoned by the researcher two weeks after receiving the information letter. They will be asked whether they have any questions and if they are interested to participate in the study. Second, the remaining four hospital sites are expected to recruit participants by a comprehensive information letter sent to them by mail. These patients will also be phoned by the researcher about two weeks after receiving the information letter to ask whether they are interested to participate in the study. When patients decide to participate, they are asked to return the included informed consent form with the reply-paid envelope within a week. Moreover, all participants will be informed about the possibility for psychological counseling nearby, so they know where to turn to in case they need (more) help.

### Randomisation

After completing the baseline measure, every patient will be randomly assigned to either the online self-help training or care as usual with an allocation ratio of 1:1. Block randomisation (block size 10) will be carried out through a sealed envelope system, for each hospital separately. Both the participants and the researchers are blinded for the randomisation process, but not for the randomisation outcome. Statistical analysis will be done by a statistician blind for randomisation outcome.

### Intervention

After conducting a survey in patients from the Dutch association of cancer patients’ organizations, the online self-help training “Less fear after cancer” was developed by the Helen Dowling Instituut, an institute for psycho-oncology in Bilthoven, the Netherlands.

**Fig. 1:**
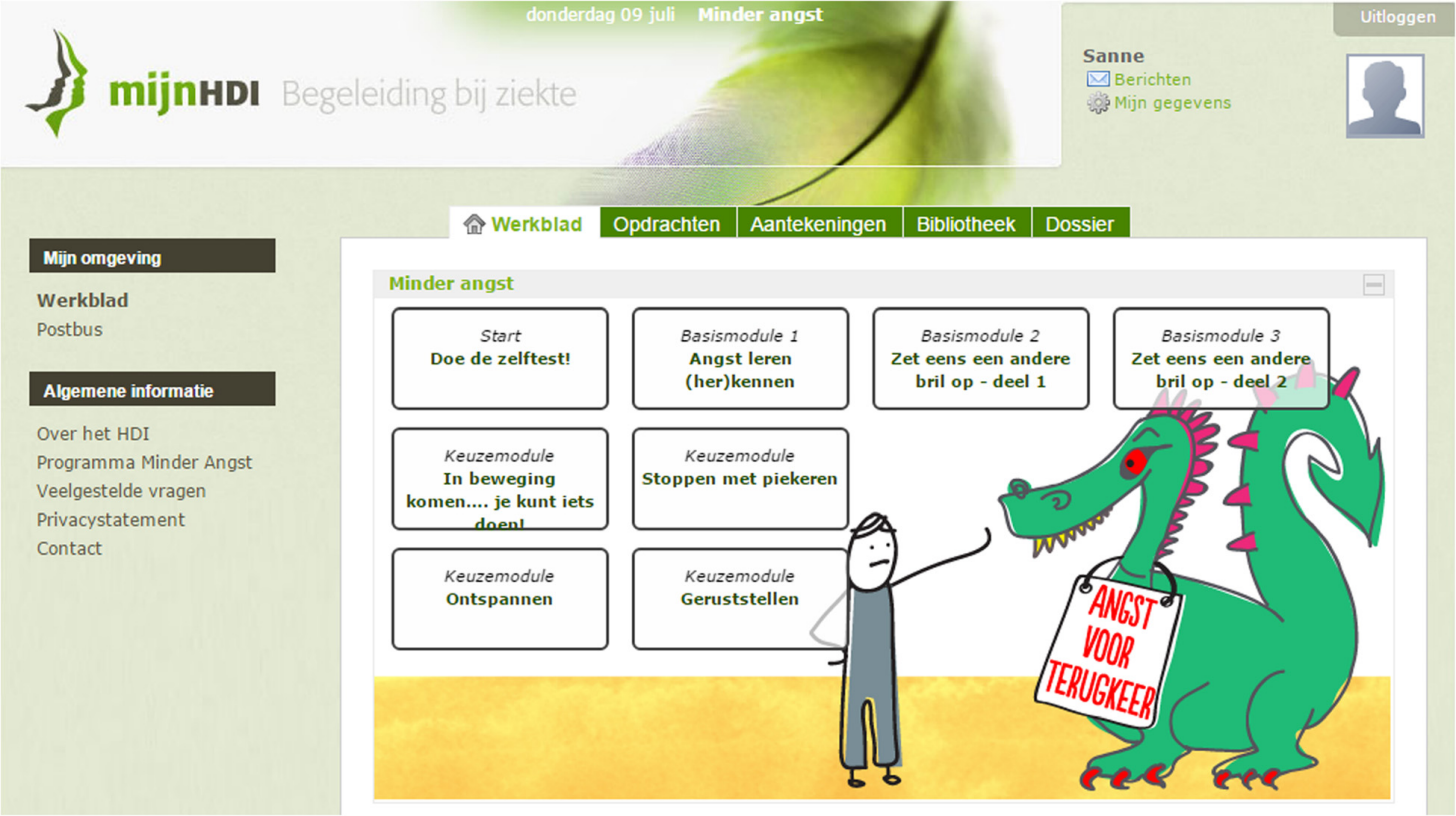
Screenshot of the “Less fear after cancer” online self-help training

“Less fear after cancer” is a tailored online self-help training based on cognitive behavioural therapy. Participants start the training by filling out the FCRI [3], after which they get (automated) feedback about their scores and a suggestion about which modules to follow. The FCRI and all modules are visible on worksheet of the intervention (see Fig. 1). First, participants follow two basic modules: 1) Psycho-education about FCR, its symptoms and learning to recognize symptoms of fear; and 2) The basic principles of cognitive behavioural therapy (this module is divided in two parts). After these basic modules women can choose from the following four the modules that are relevant to their situation: 1) How to stop rumination, behavioural techniques to stop ruminating; 2) Action, making an action plan about what one can do when fear of recurrence pops up; 3) Relax, audio files with relaxation practices; and 4) Reassurance, how and when to seek reassurance. Each module consists of an informative part and a practical part in which participants are motivated to do exercises or assignments in daily life. Participants are advised to take a week for each module they choose, so most participants will need four to six weeks depending on how many modules they do. It is explained that the more time they invest, the more effect they can expect from the training, but participants eventually choose themselves how much time is actually spent on the training. For every patient, the intervention will be available for three months.

The most important functionality of the online self-help training “Less fear after cancer” is the worksheet, because it gives an overview of the modules and access to the intervention. By clicking on a module, participants can access the information (texts, videos, audio files) and exercises of the self-help training. Other functionalities include a library with the information and forms in pdf format, videos, audio files, and a mailbox for technical assistance.

“Less fear after cancer” is fully automated and primarily non-guided and is delivered without professional support from a therapist. In this study, personal online support by an e-mail coach (the researcher) is available for the women that indicate a need for this. The e-mail coach can give technical assistance and eventually refer participants to their general practitioner or medical specialist when they indicate a need for professional help.

### Usual care

The control group of this RCT has access to usual care. Care as usual may differ somewhat between hospitals and may include psychosocial care from within the hospital or elsewhere. In the Medical Consumption Questionnaire, use of psychosocial care will be assessed. Care as usual will be available in both conditions, in the intervention condition the online self-help is extra.

### Outcomes

All participant outcomes will be gathered using online self-report questionnaires hosted by SurveyMonkey.com. Participants will receive an invitational e-mail with a link to complete the questionnaires online. The questionnaires at baseline, 3 months, 9 months, and 24 months include questions on socio-demographic and medical variables.

### Primary outcomes

*Fear of cancer recurrence* will be assessed using the 43-item Dutch version of the FCRI [3]. The FCRI consists of statements rated on a 5-point Likert scale ranging from 0 (not at all or never) to 4 (a great deal or all the time). The FCRI includes seven subscales: triggers, severity, psychological distress, coping strategies, functioning impairments, insight, and reassurance. The triggers-subscale evaluates the presence of potential stimuli activating FCR. Psychological consequences of FCR are evaluated by the subscales psychological distress and functioning impairments. The insight scale measures the level of self-criticism towards FCR intensity. The reassurance- and coping strategies-scales measure a variety of coping strategies than can be used to cope with FCR including denial, wishful thinking, cognitive avoidance, and reassurance. The severity subscale assesses the presence and severity of intrusive thoughts or images associated with FCR and this scale can be used separately as a brief screening instrument of FCR and as an outcome measure [3]. The severity subscale is the primary outcome measure. The coping strategies- and functioning impairments-scale scores at baseline will be used in the predictor analysis. The original 42-item French-Canadian version of the FCRI had a good internal consistency (Cronbach’s α = 0.95 for the total score and α = 0.89 for the severity subscale) and stable over a 1-month interval (*r* = 0.89, *p* < 0.001) [3]. The scale has a robust factor structure and the results support construct validity with other self-report scales assessing FCR (r’s 0.68 to 0.78) or related constructs (r’s 0.43 to 0.66) and quality of life (r’s −0.20 to −0.36) [3]. The Dutch version of the FCRI (FCRI-NL) is currently being validated.

*Fear of cancer recurrence* will also be assessed with the Dutch version of the Cancer Worry Scale (CWS) [43]. The CWS assesses concerns about developing cancer or developing cancer again and the impact of these concerns on daily functioning. The Dutch version of the CWS consists of 8 items that are rated on a 4-point Likert scale ranging from 1 (never) to 4 (always). Higher scores indicate more frequent worries about cancer. A cut-off score of 13 (low ≤13, high ≥14) turned out to be optimal for detecting severe levels of FCR [44]. Moreover, the CWS is a reliable questionnaire (Cronbach’s α = 0.87) and evidence has been found to support the construct validity [44].

### Secondary outcomes

*Healthcare costs* will be assessed with the Medical Consumption Questionnaire (MCQ), a questionnaire to assess non-disease specific healthcare costs [45]. More precisely, the volume of used healthcare will be assessed with the MCQ. Afterwards, the Dutch Manual on Cost Investigations will be used to calculate the healthcare costs [46].

Furthermore, the Dutch translation of the EuroQol-5D (EQ-5D), a generic measure of health status, will be used for the economic evaluation [47, 48]. The EQ-5D comprises five domains: mobility, self-care, usual activities, pain/discomfort, and anxiety/depression. Each domain consists of one question with three answer categories: (1) no problems, (2) some problems, and (3) extreme problems [47]. A health state can be derived by combining the scores from each dimension [47]. This results in a 5-digit number, for example state 12233. This health state indicates no problems with mobility, some problems with self-care and usual activities, and extreme problems with pain/discomfort and anxiety/depression [47, 48]. EQ-5D health states may be converted into a EQ-5D index by applying predetermined weights to the five domains [49]. The Dutch EQ-5D tariff will be used to value this EQ-5D index [47]. The EQ-5D index gives a societal-based global quantification of the patient’s health status on a scale ranging from 0 (death) to 1 (perfect health) [50]. For economic evaluation, the EQ-5D index scores will be used to determine quality-adjusted life years (QALYs) [50]. Patients will also be asked to rate their overall health status on a visual analogue scale (EQ-5D VAS), a quantitative self-rating of health status in which patients are asked to rate their current health state on a 0 (worst imaginable health status) to 100 (best imaginable health status) scale [48]. The EQ-5D is a ‘user-friendly’ questionnaire, with acceptable reliability and validity for various populations [51–53].

*Psychological distress* will be assessed with the corresponding subscale of the FCRI-NL [3].

### Process outcomes

In addition to the self-report questionnaires, technical data on the use of the intervention will be gathered in the intervention group. For example, frequency of logins, duration of logins, and website activity will be evaluated.

### Other outcomes

The *use of extra (psychological) help* will be assessed with the Medical Consumption Questionnaire (MCQ) [46]. Furthermore, help or referral by the e-mail coach will be registered and added to the MCQ score.

*Psychosocial problems* and risk factors will be assessed with the Psychosocial Distress Questionnaire-Breast Cancer (PDQ-BC) [54]. The PDQ-BC is a multi-dimensional screening instrument specific for breast cancer patients. It consists of nine subscales using 35 items assessing psychological risk factors (i.e. trait anxiety and (lack of) social support) and state anxiety, depressive symptoms, social problems, physical problems, body image, financial problems, and sexual problems. All items are answered on a 4-point Likert scale, ranging from 1 (not at all) to 4 (very much) [54]. For most subscales, high scores indicate more psychosocial problems, except for body image and social support for which higher scores indicate fewer problems [55]. The PDQ-BC appears to have a sufficient internal consistency, and good construct validity, test–retest reliability, and sensitivity to change. Furthermore, the PDQ-BC subscales state anxiety and depressive symptoms have a satisfactory sensitivity and specificity [54–56].

*Self-efficacy for online self-help* will be assessed with a questionnaire which was especially assembled for the current study. Bandura [57] argued that all-purpose measures of perceived self-efficacy usually have limited explanatory and predictive value because most of the items in an all-purpose test may have little or no relevance to the domain of functioning. Scales of perceived self-efficacy should be tailored to the particular domain of functioning that is the object of interest. Therefore, we collected many potentially useful items from various self-efficacy questionnaires [58–62]. In consultation with both professionals and patients, we improved and reduced the items to a 15 item questionnaire tailored to assess self-efficacy for our online self-help training. The items are rated on a 5-point Likert scale, ranging from 1 (not like me) to 5 (totally like me). The items are divided in three domains: 1) general internet use (3 items); 2) health related coping strategies (7 items); and 3) patients’ expectations on online self-help training for fear of cancer recurrence (5 items).

A subsample of patients (*n* = 16) will be asked about their experience with the online self-help in a semi-structured interview, to evaluate the online self-help training and to detect possible ways to further improve the training. There are different profiles of FCR, which vary according to its severity and the type of coping strategies used. Patients will be selected based on their baseline score on the FCRI and will represent different FCR-profiles: mild FCR-severity and low coping, mild FCR-severity and high coping, moderate FCR-severity and high coping, moderate FCR-severity and low coping [63]. From the first 50 participants who finished the online self-help training, four participants from each FCR-profile group will be randomly picked. If women refuse to participate, another participant will be randomly picked from that group.

### Sample size calculation

The sample size calculation is based on a clinically relevant improvement on the FCRI severity subscale at 3 months. With an effect size of *d* = 0.3, a minimum number of 2.28 points on the FCRI severity subscale could be detected. Based on our experience with the FCRI, this seems to be a clinically relevant difference. In total 454 patients need to be included (227 in each group) to statistically detect the minimum effect size of *d* = 0.3 between mean FCRI severity subscale scores of both groups with a power of 0.8 and a two-sided alpha of 0.05. The power analysis program G*Power 3.1.7 was used to calculate the effect sizes [64]. Since in previous online intervention studies amongst breast cancer patients about half of all invited patients expressed an interest in participating and another 35 % was lost after randomization [65, 66], the aim of this study is to ask a minimum of 900 patients to participate in the study.

### Statistical analysis

#### Primary analyses

Baseline characteristics in both groups will be compared to check if randomisation has resulted in an equal distribution of the baseline variables. Data will be analysed according to the intention-to-treat (ITT) principle. In the primary analyses, post-training scores will be compared between the two groups, controlling for baseline symptom levels. Analyses will be performed using t-tests and linear mixed models including exploratory predictor analyses. An advantage of linear mixed models is the optimal use of available data. The primary analysis is aimed at comparing the online self-help with care as usual at 3 months on the FCRI severity subscale. Analysis of co-variance will be performed to test whether the outcome variables differed between the online self-help and care as usual, using baseline level as covariate. The stability of the results will be analysed using the data from 9 months and 24 months after baseline, again using linear mixed models to control for the dependency caused by the repeated measurements. Time will be analysed as a categorical predictor with four levels (baseline - 3 months - 9 months - 24 months). Linear mixed models with a specified covariance pattern model will be used to examine the course of FCR [67]. The fixed-effects parameters of the models will be estimated with maximum likelihood. Inspection of the Log likelihood ratio test, Akaike Information Criterion (AIC), and Bayesian Information Criterion (BIC) with restricted maximum likelihood (REML) will be used to find most suitable covariance pattern model (e.g., compound symmetry, autoregressive, unstructured).

For the secondary analyses, significant predictors will be selected using exploratory predictor analyses. Then multivariate hierarchical regression analysis will be performed to assess predictors of effect. The predictors that are found statistically significant will be added as interaction terms with condition (online self-help versus care as usual). Drop outs (attrition) will be closely investigated and predictors of drop outs will also be analysed by multivariate regression analysis.

#### Cost-effectiveness analyses

The economic evaluation will consist of a cost-effectiveness analysis and a cost-utility analysis, both done from a societal perspective [50]. The cost-effectiveness ratio will represent the costs per significantly improved participant, while the cost-utility analysis will represent the costs per additional quality-adjusted life year (QALY). The time horizon will be life time. As the study period is limited in time, cost and effect will be modelled in time, using the assumption that the spontaneous recovery is 2 years. The effects of this assumption on the cost-effectiveness ratio will be tested by testing the scenarios of spontaneous recovery after 6 months, 1 year and 5 years.

Costs will be estimated from a societal perspective and will thus include the costs related to the intervention, all other healthcare costs and non-healthcare costs during the time horizon of the study. Healthcare consumption will be measured with the MCQ. Healthcare consumption includes all non-disease specific healthcare used in the previous 3 months, such as visiting the general practitioner or other healthcare providers, emergency room visits, hospitalisation, and medication use. Then, the guidelines as descripted by the Dutch Manual on Cost Investigations will be used to calculate the healthcare costs [46]. For healthcare where no guideline or standard prices are available, real cost prices will be determined or, when available, derived from the health care provider administration.

QALYs will be calculated from EQ-5D health states using the Dutch EQ-5D tariff [47]. Non-parametric bootstrap simulations will be used to estimate uncertainty intervals around the ICERs, in order to deal with the most likely skewed distributions of costs. Cost-effectiveness acceptability curves will be calculated to show the probability that the intervention is cost-effective in comparison with the control group, given varying thresholds for the willingness-to-pay for gaining one unit of effect, i.e. a QALY or a significantly improved participant. The robustness of the results will be explored using one-way sensitivity analyses in which the input variables for assessing both cost and effectiveness are varied.

## Discussion

FCR is one of the most common long-term consequences of surviving cancer, with a major impact on personal, family, and professional life and is associated with considerable healthcare costs. In order to ensure the availability of affordable evidence-based psychological interventions for FCR in the future, research on cost-effective interventions for FCR is needed. This is especially relevant for psychological care in breast cancer survivors, as breast cancer is the most prevalent form of cancer in women and the prevalence is still increasing, together with the healthcare costs [1]. Online self-help training may be an efficient and cost-effective way to offer psychological treatment for this group. Moreover, online self-help may provide easy-accessible treatment for the large group of breast cancer survivors with low to moderate FCR, diminishing the number women in need of face-to-face therapy. Therefore, the CAREST-study has been designed to evaluate the effectiveness and cost-effectiveness of an online self-help training for FCR.

### Strengths and weaknesses

In the proposed study, an online CBT-based self-help training will be compared with care as usual. To the best of our knowledge, this is the first study to evaluate an online self-help training for FCR in breast cancer survivors. Using the internet, this intervention provides a novel and easy-accessible approach to reduce the impact of FCR at an early stage. In the long term this study may contribute to early prevention of FCR. Moreover, a health economic evaluation is included in the trial, to assess the cost-effectiveness of the online self-help training up to 24 months. The 24 months follow-up period strengthens the study, whereas many studies only include follow-up periods up to 12 months. The results of the study will provide information on the possible strengths and benefits of online self-help training for FCR in breast cancer survivors. However, since both the intervention and the control group are allowed to receive usual (psychological) care during the study, the expected effects are small. Inclusion of women with all levels of FCR, ranging from no FCR to considerable FCR, may also decrease the effectiveness of the online self-help training. Nevertheless, this design provides the opportunity to predict who benefits most from the intervention. Therefore, this trial will provide clinically relevant information. Considering the lack of intervention research for FCR in breast cancer patients, this study will contribute to the body of knowledge about how to best support this large and growing group. Research into alternative, long-term effective treatment options for this group will be of great clinical value. The eventual goal of the proposed study is to implement an easy accessible, evidence-based and cost-effective intervention for FCR in women who benefit most from these kind of online self-help interventions, within the follow-up care of breast cancer patients in the Netherlands.

## Trial status

Inclusion has started in April 2014. Inclusion is estimated to take up to two years, with one to two years follow-up. Final results are expected around summer 2017.

## Abbreviations

ACT, Acceptance and Commitment Therapy; AIC, Akaike Information Criterion; BIC, Bayesian Information Criterion; CAREST, CAncer REcurrence Self-help Training; CBT, Cognitive behavioural therapy; CMO, Medical Ethics Committee; CONSORT, Consolidated Standards of Reporting Trials; CWS, Cancer Worry Scale; EQ-5D, EuroQol-5D; FCR, Fear of Cancer Recurrence; FCRI, Fear of Cancer Recurrence Inventory; FCRI-NL, Dutch version of the Fear of Cancer Recurrence Inventory; ITT, intention-to-treat; MBCT, Mindfulness Based Cognitive Therapy; MBSR, Mindfulness Based Stress Reduction; MCQ, Medical Consumption Questionnaire; PDQ-BC, Psychosocial Distress Questionnaire-Breast Cancer; QALY, quality-adjusted life year; RCT, Randomised Controlled Trial; REML, restricted maximum likelihood; SPIRIT, Standard Protocol Items: Recommendations for Interventional Trials; VAS, visual analogue scale
